# Large-Scale Fabrication of Boron Nitride Nanotubes via a Facile Chemical Vapor Reaction Route and Their Cathodoluminescence Properties

**DOI:** 10.1007/s11671-010-9794-8

**Published:** 2010-09-26

**Authors:** Bo Zhong, Xiaoxiao Huang, Guangwu Wen, Hongming Yu, Xiaodong Zhang, Tao Zhang, Hongwei Bai

**Affiliations:** 1 School of Materials Science and Engineering, Harbin Institute of Technology, 150001 Harbin, People's Republic of China; 2 School of Materials Science and Engineering, Harbin Institute of Technology (Weihai), 264209 Weihai, People's Republic of China

**Keywords:** Boron nitride nanotubes, Luminescence performance, Growth mechanism, Ammonia borane

## Abstract

Cylinder- and bamboo-shaped boron nitride nanotubes (BNNTs) have been synthesized in large scale via a facile chemical vapor reaction route using ammonia borane as a precursor. The structure and chemical composition of the as-synthesized BNNTs are extensively characterized by X-ray diffraction, scanning electron microscopy, high-resolution transmission electron microscopy, and selected-area electron diffraction. The cylinder-shaped BNNTs have an average diameter of about 100 nm and length of hundreds of microns, while the bamboo-shaped BNNTs are 100–500 nm in diameter with length up to tens of microns. The formation mechanism of the BNNTs has been explored on the basis of our experimental observations and a growth model has been proposed accordingly. Ultraviolet–visible and cathodoluminescence spectroscopic analyses are performed on the BNNTs. Strong ultraviolet emissions are detected on both morphologies of BNNTs. The band gap of the BNNTs are around 5.82 eV and nearly unaffected by tube morphology. There exist two intermediate bands in the band gap of BNNTs, which could be distinguishably assigned to structural defects and chemical impurities.

## Introduction

As structural analogs of carbon nanotubes (CNTs), boron nitride nanotubes (BNNTs) have attracted continuous attention owing to their extraordinary structural and physical properties [1,2]. Similar to CNTs, BNNTs possess a superior Young's modulus and a high thermal conductivity [3-6]. BNNTs are electrical insulator and transparent to visible light due to a wide band gap (around 5.2–5.8 eV) that is almost independent of tube chirality [7,8]. Furthermore, BNNTs exhibit excellent chemical stability and inoxidizability [2,9]. The unique structure-induced properties of BNNTs bring a series of opportunities for their potential applications as hydrogen storage media, biological probes, piezoelectric materials, composite reinforcements and harsh-environment semiconductor devices [10-17]. These promises have motivated intense research efforts seeking to develop synthetic strategies for preparing BNNTs.

Despite the structural similarity between BNNTs and conventional CNTs, great challenges have been encountered in fabricating BNNTs compared with the relative ease of synthesizing CNTs. Many techniques, such as arc-discharge [1], ball milling and annealing [18-21], laser ablation [22-24], chemical vapor deposition [25,26], oven heating proper B and N containing precursors [27,28], template confining [29,30], and so forth, have been attempted to fabricate the BNNTs in recent years. Although some success has been achieved in producing pure and well-crystallized BNNTs, these techniques generally require special equipments or complex synthesis procedures and the yields of BNNTs are commonly disappointingly low.

Here, we describe a facile growth technique that can easily and reliably produce macroscopic amounts (~200 mg per experimental run) of BNNTs with cylinder and bamboo-shaped morphologies. Ammonia borane (AB, H_3_BNH_3_), which contains only B, N and H elements, is demonstrated to be an effective starting material for the fabrication of BNNTs. The structures and luminescence performance of the as-synthesized BNNTs have been extensively characterized. A two-step growth model has been established based on the analysis of the structures of BNNTs and the reaction process. The present work provides a facile synthetic approach and a deeper insight into the luminescence performance of BNNTs, which facilitates large-scale production of BNNTs and their application as compact ultraviolet (UV) laser devices.

## Experimental

In this study, we present a simple approach for the fabrication of BNNTs in a gas pressure furnace. Ammonia borane (AB) synthesized according to Ramachandran [31] was used as a starting material, and ferrocene was used as a catalyst. In a typical procedure, AB powder (4.0 g) and ferrocene (1.5 g) were mixed and charged into an graphite crucible of about 2 l capacity using a piece of graphite paper as inner lining, then the crucible was loaded into the furnace chamber. The chamber was sealed and pumped down to a base pressure of 0.1 Pa. Subsequently, 0.8 MPa high pure nitrogen was pressed into the furnace chamber. The furnace was heated to 1,450°C at a rate of 10°C min^-1^ and held for 60 min before it was finally cooled to room temperature. The BNNTs were found on the graphite paper. The samples obtained were extensively characterized by scanning electron microscopy (SEM, MX2600EF equipped with energy dispersive X-ray spectroscopy (EDX)), transmission electron microscopy (TEM, Philips Tecnai 20 and Tecnai F30 FEG equipped with electron energy loss spectroscopy (EELS)), X-ray powder diffraction (XRD, Rigaku D/max-γB X-ray diffractometer with Cu K radiation (λ = 0.154178 nm)), X-ray photoelectron spectroscopy (XPS, PHI 5700 ESCA System with a PC-ACCESS data analysis system (Physical Electronics Inc.)), Fourier transformation infrared spectroscopy (FTIR, Perkin Elmer spectrum one system by using pressed KBr disks) and ultraviolet–visible spectroscopy (Perkin-Elmer Lambda 950 UV/Vis Spectrophotometer). Cathodoluminescence (CL) measurements were performed using a Gatan MONOCL3 + system installed on a JSM-7000F SEM.

## Results and Discussion

**Figure 1:**
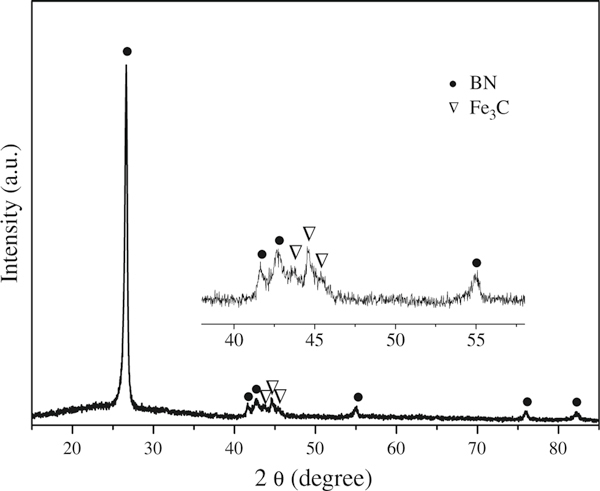
**XRD pattern of the as-synthesized BNNTs**.

Figure 1 presents a XRD pattern of the as-grown products, from which two crystalline phases could be indexed: hexagonal BN (h-BN, JCPDS 34-0421) and Fe_3_C (JCPDS 35-0772). The dominant BN phase suggests that the products are mainly composed of h-BN. The Fe_3_C phase is apparently related to the catalyst, which will be further discussed in later sections.

**Figure 2:**
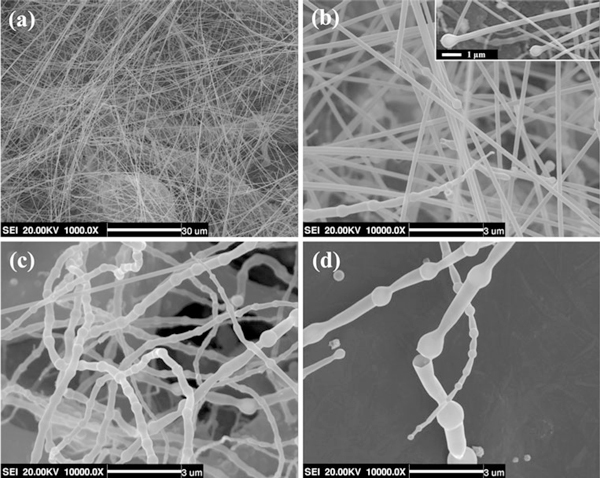
**SEM images of the as-synthesized BNNTs showing a overall morphology, b cylinder-shaped BNNTs, c bamboo-shaped BNNTs and d a broken site of the bamboo-shaped BNNTs**.

Figure 2 shows representative SEM images of the as-synthesized products. A low magnification SEM image shown in Figure 2a suggests that a high yield of one-dimensional (1D) nanostructures is obtained. High magnification SEM images further reveal that the products actually consist of two morphologies of 1D nanostructures. One is the cylinder-shaped nanostructures with typical diameters ranging from 15 to 200 nm and lengths up to hundreds of microns (Figure 2b). The cylinder nanostructures commonly contain knobs on their tips, as shown in inset of Figure 2b. The other is the bamboo-shaped nanostructures with diameters around 300 nm and length up to tens of microns (Figure 2c). The latter type of nanostructure is decorated by periodically appeared knobs. It is interesting that some bamboo-shaped nanostructures expose their broken sites, which provides direct evidence that the bamboo-shaped nanostructures are tubes, as shown in Figure 2d.

**Figure 3:**
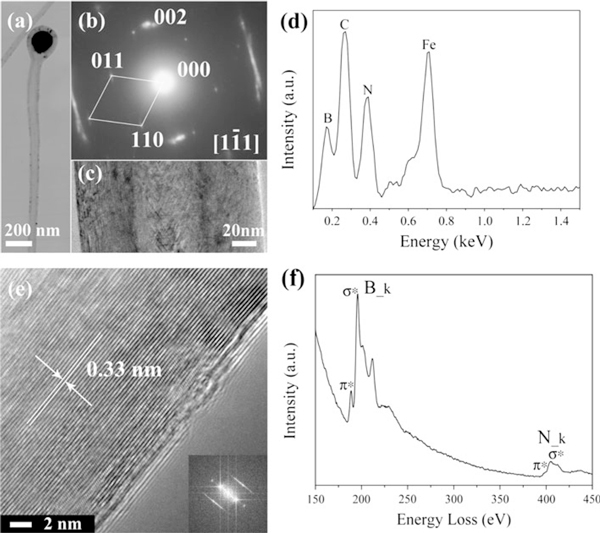
**a A bright-field TEM image showing a segment of cylindrical BNNT**. **b** SAED pattern taken from catalyst-containing knob on the tip of a cylindrical BNNT. **c** High magnification TEM image of the tubular part of a cylindrical BNNT. **d** EDX spectrum collected from a catalyst-containing knob on the tip of a cylindrical BNNT. **e** HRTEM image taken from the tube wall of a cylindrical BNNT, inset: the fast Fourier transform pattern of the corresponding HRTEM image. **f** EELS spectrum of tubular part of a cylindrical BNNT.

The structure and chemical composition of the as-synthesized 1D nanostructures were further characterized using TEM, SAED, HRTEM, EDX and EELS. Figure 3a displays a typical bright-field TEM image of the cylinder-shaped nanostructures, showing a perfect hollow-cored cylindrical nanostructure with a particle on its tip. A high magnification TEM image of the tubular part of the nanostructure shown in Figure 3c further confirms the hollow nature. The EELS spectrum collected from the tubular part of the nanostructure is depicted in Figure 3f. The pronounced peaks located at about 188 and 401 eV correspond to the characteristic K-shell ionization edges of B and N atoms, respectively. The 284 eV carbon K edge is notably absent, revealing that the cylindrical nanostructure is free of carbon. The approximate atomic ratio B:N is determined to be around 1.0 within the experimental error, exhibiting the expected chemical composition of BN. Combined TEM and EELS analyses indicate that the cylinder-shaped 1D nanostructures observed in SEM images are BNNTs. The detailed structure of the cylindrical BNNTs was further investigated by HRTEM. Figure 3e displays a HRTEM image of the tube wall of an individual cylindrical BNNT. Ordered lattice fringes with identical spacing of about 0.33 nm are clearly identified, corresponding to the (002) planes of h-BN. This suggests that the tube walls are composed of well-crystallized BN. The fast Fourier transform (FFT) pattern of the HRTEM image also confirms a highly crystalline nature (see inset in Figure 3e).

The particle on the tip of the cylindrical BNNT is believed to be the catalyst that catalyzes the growth of the nanotube. A representative SAED pattern taken from the particle is shown in Figure 3b, which can be indexed as that taken along the [1-11] zone axis of a Fe_3_C crystal (JCPDS 35-0772). Sharp spots from the (002) plane of h-BN (JCPDS 34-0421) are also clearly observed, which apparently originate from the BN walls encapsulating the particle. It is noteworthy that no spots could be indexed to α-Fe, implying that no α-Fe phase is encapsulated in the tip. The EDX spectrum acquired from the tip of a cylindrical BNNT is displayed in Figure 3d, which clearly shows the strong signals from Fe and C, together with B and N. The EDX result further demonstrates that the metal particles encapsulated in the tips of BNNTs are Fe_3_C rather than simple substance α-Fe, which is in agreement with the SAED analysis.

**Figure 4:**
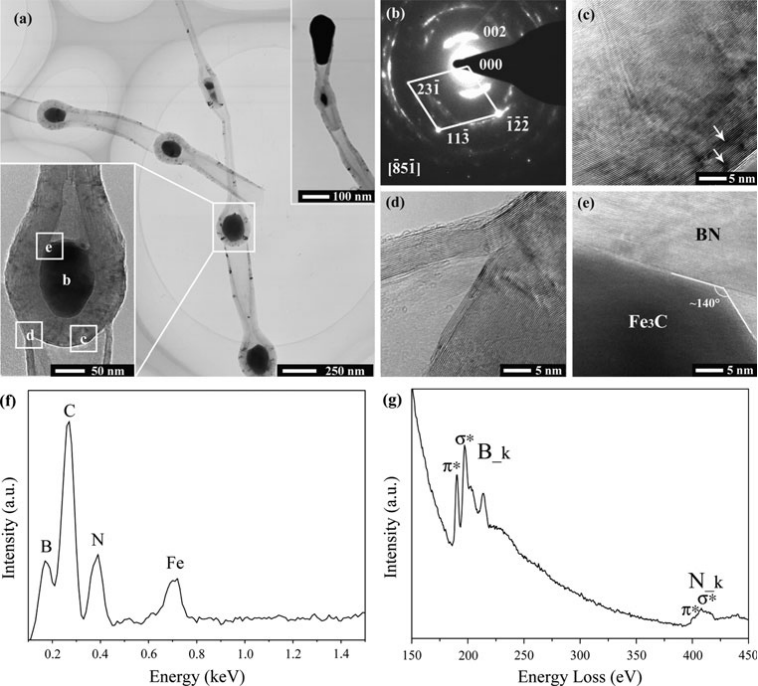
**a A typical bright-field TEM image showing the bamboo-shaped BNNTs, upper inset: a representative tip reflecting catalyst extruding; lower inset: a high magnification TEM image of a typical knob**. **b** SAED pattern taken from a catalyst-containing knob marked in part a. HRTEM images taken from the areas framed in part a showing: **c** defects containing BN walls of a knob encapsulating a catalyst particle, **d** detailed structure of the joint connecting two bamboo units and **e** interface between BN sheets and a catalyst particle. **f** EDX spectrum of the knobs of bamboo-shaped BNNTs. **g** EELS spectrum collected from tubular part of a bamboo-shaped BNNT.

Figure 4a displays a typical bright-field TEM image of the bamboo-shaped 1D nanostructures. Tubular nanostructures formed via repetition of a series of bamboo units are clearly observed. The EELS spectrum of the tubular part of bamboo-shaped nanostructures is shown in Figure 4g. Similar to that of the cylindrical BNNTs, peaks originating from B and N elements are clearly identified.

Quantitative analysis also gives a stoichiometric composition of BN, indicating that the bamboo-shaped 1D nanostructures are BNNTs. Although some empty knobs are occasionally observed, most knobs contain uniform quasi-spherical catalyst particles inside. These catalyst particles are confirmed to be Fe_3_C by SAED pattern, as shown in Figure 4b. Similar to that of cylindrical BNNTs, α-Fe phase was not detected in the knobs of bamboo-shaped BNNTs. EDX analysis was also performed to verify the chemical composition of the particles in the knobs, as shown in Figure 4f, which also confirms that the particle is Fe_3_C. Bamboo-shaped BNNTs containing α-Fe in their knobs has been reported previously [28], but in our case, the particles contained in the knobs of the BNNTs are Fe_3_C instead of α-Fe, which might imply a different formation mechanism of the bamboo-shaped BNNTs. It is noted that the catalyst that catalyzed the growth of both BNNTs is Fe_3_C rather than α-Fe. This is beneficial to the low-temperature growth of BNNTs since the melting point of Fe_3_C is much lower than that of α-Fe.

Several interesting features of the bamboo-shaped BNNTs are evidenced by HRTEM analysis. Figure 4c shows a HRTEM image of a BN shell that encapsulates a catalyst particle. Some dark regions originating from edge dislocations could be clearly observed, especially in zones far from the catalyst, as marked by arrows. These dislocations are caused by inherent strains [32], implying the existence of stresses in the outer BN layers. HRTEM image displayed in Figure 4d suggests that the junctions of bamboo units are formed by continuous BN layers and the bamboo units are tightly linked. Figure 4e shows a typical HRTEM image of the interface between a catalyst particle and the tube wall, revealing a poor compatibility between the catalyst particle and the inner wall of the BNNTs since the contact angle is about 140 degrees (much larger than 90 degrees). The poor compatibility facilitates the growth of hollow BNNTs without catalysts filling because molten catalyst particle with a high surface tension tends to fuse together and move as a whole.

**Figure 5:**
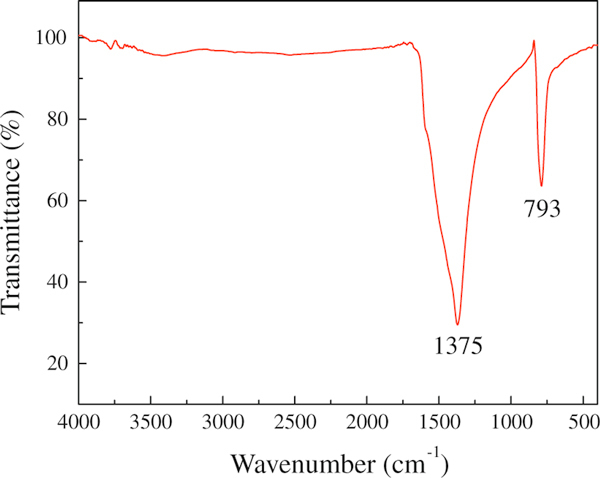
**FTIR spectrum of the as-synthesized BNNTs**.

The as-synthesized products containing two morphologies of BNNTs were analyzed in transmittance mode by FTIR to investigate their bonding nature. The FTIR spectrum displayed in Figure 5 shows strong vibrations at 1,375 and 793 cm^-1^, which are characteristic primary and secondary absorption bands of h-BN, respectively. The absorption band at 1,375 cm^-1^ is associated with the well-known in-plane vibrational mode (E_2g_) of the h-BN networks, while the weak absorption bands at 793 cm^-1^ corresponds to the out-of-plane vibration [33]. Both the spectrum profile and the absorption bands are similar to that reported for BNNTs, which further confirms that the as-synthesized products are BNNTs.

**Figure 6:**
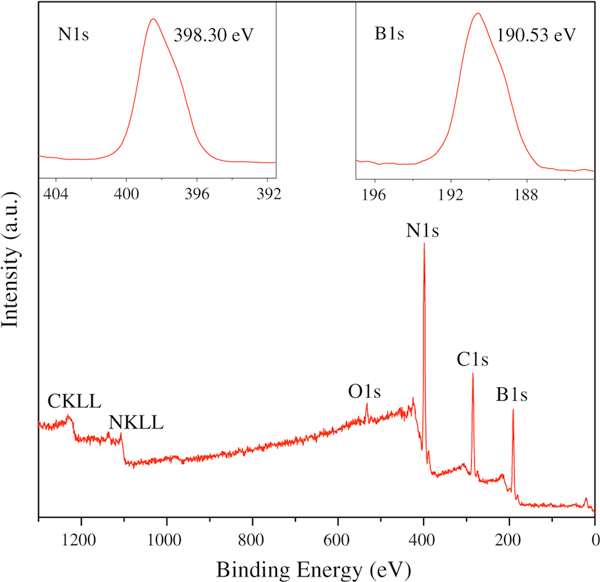
**Wide scan, N1s (inset: *upper left*) and B1s (inset: *upper right*) XPS spectra recorded from the as-synthesized BNNTs**.

XPS analysis additionally indicates that the as-prepared products contains N and B elements, as shown in the full range survey XPS spectrum displayed in Figure 6. The high-resolution N 1s and B 1s spectra depicted in the insets of Figure 6 are nearly of Gaussian type and are not decomposed further. The N 1s peak at binding energy near 398.30 eV and the B 1s peak at binding energy around 190.53 eV can be assigned to the N and B atoms in h-BN, respectively [34]. The carbon and oxygen signals observed in the XPS profile are ascribed to the surface contamination during the exposure of the sample to the air before the examination. The B/N atomic ratio calculated according to the XPS spectra is around 0.9. Both the binding energies and the atomic ratio of boron and nitrogen atoms are close to that of h-BN, implying that the as-prepared products are BNNTs.

To gain a better understanding of the growth mechanism of the BNNTs, a variety of controlled experiments were carried out. It is found that BNNTs could be obtained within a wide temperature range of 900–1,600°C (see Figure S1 in supporting information). The highest yield is achieved at about 1,450°C. BNNTs could also be obtained under argon atmosphere, but the yields are generally low (see Figure S2 in supporting information). When the amount of ammonia borane is increased, diameters of the BNNTs are dramatically enlarged and their structures are ultimately altered (see Figure S3 in supporting information). This is attributed to the high concentration of B and N containing gases, which may accelerate the growth rate of BNNTs and result in the structural alteration.

**Figure 7:**
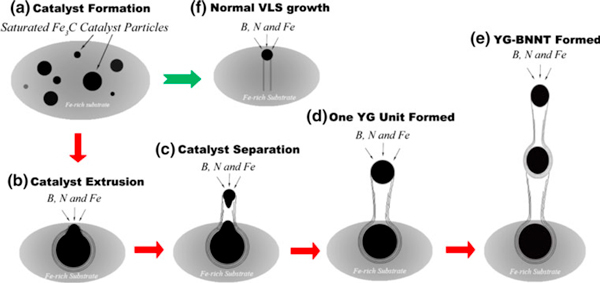
**Schematic illustration of the formation processes of a–e bamboo-shaped BNNTs and f cylindrical BNNTs**.

On the basis of the above characterizations and our experimental procedure, the formation process of the two types of BNNTs can be rationally expressed as a two-stage growth mechanism. The evolving process of each type of BNNTs is schematically illustrated in Figure 7. The growth of the BNNTs involves the following two stages: the formation of catalyst particles oversaturated with B and N atoms, and the precipitation of BN sheets on the catalyst particles via migration of the B and N atoms to the catalyst surface [35]. During the first stage, the ferrocene decomposes according to the following reaction [36], Fe(C_5_H_5_)_2_ → Fe + H_2_ + CH_4_ + C_5_H_6_ + ..., forming iron particles and carbon-containing vapors. On the other hand, ammonia borane decomposes based on the following route, BH_3_NH_3_ → H_2_ + BN_*x*_H_*y*_, where BN_*x*_H_*y*_ indicates gaseous residues containing B and N, including monomeric aminoborane (BH_2_NH_2_), borazine (BHNH)_3_ and traces of diborane (B_2_H_6_) [37-40]. These vapors might adsorb onto the melted or partially melted iron-particle surfaces and decompose into C, B and N atoms. This would result in catalyst particles supersaturated with B and N atoms, as shown in Figure 7a.

As soon as the supersaturated catalyst particles are formed, BN layers begin to precipitate from the catalyst particles onto the surfaces. At this juncture, BNNTs are likely to grow in different scenarios because the precipitation rate of B and N atoms might depend on the curvatures of the catalyst particles, similar to that occurs in the growth process of CNTs [41,42]. BN sheets on smaller catalyst particles exhibit larger curvatures, and their formation are energetically unfavorable. Thus, the precipitation of B and N atoms from smaller catalyst particles is relatively slow. The B and N atoms have enough time to locate themselves onto favorable sites, i.e., the edges of the formerly formed BN sheets, which would push the catalyst particles on the tube tips to go ahead and facilitate the growth of the C-BNNTs, as shown in Figure 7f. This is essentially the well-known vapor–liquid–solid (VLS) growth process [43]. On the contrary, the surfaces of the larger catalyst particles are more flat, and the precipitation of the B and N atoms on such surfaces are more energetically favorable. The BN sheets precipitate at such a high rate that they form a BN shell around the catalyst particles. The newly formed BN layers between the formerly formed BN shells and the molten catalyst particle would exert a stress on both sides, which may extrude the molten catalyst particle outside of the BN shell (Figure 7b). During the extruding process, the catalyst particle continues to adsorb source vapors and precipitate BN layers forming the tubular part of the bamboo-shaped BNNTs, as shown in Figure 7c and upper inset of Figure 4a. Consequently, the catalyst particle is present at the open end of the bamboo unit, and the growth of the following generation of bamboo unit will continue, as illustrated in Figure 7d, e.

**Figure 8:**
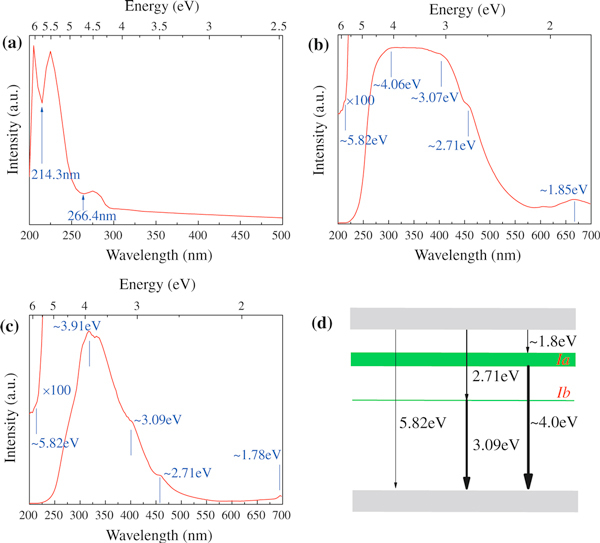
**a Ultraviolet–visible spectrum of the as-grown BNNTs (reflection mode)**. Cathodoluminescence spectra recorded from the tubular parts of **b** bamboo-shaped BNNTs and **c** cylindrical BNNTs at room temperature. **d** A schematic energy level diagram of BNNTs.

The luminous absorption property and the band gap of the as-grown BNNTs were investigated by UV/Vis spectroscopy operating in a reflection mode, as shown in Figure 8a. The ultraviolet–visible spectrum exhibits two absorption bands at 266.4 nm (4.65 eV) and 214.3 nm (5.79 eV), corresponding to the excitonic absorption and optical band-edge absorption of BNNTs [44]. The band gap of our BNNTs is comparable to that reported in the literature and is extremely close to the band gap of h-BN [33,45,46].

To investigate the luminescence performances of bamboo-shaped BNNTs and cylindrical BNNTs, cathodoluminescence (CL) analyses were performed on the tubular parts of both BNNTs, as displayed in Figure 8b, c. Both spectra exhibit similar UV emission peaks, suggesting that the BNNTs could be potentially used as compact UV laser emitters. These emission peaks could be understood by virtue of an energy level diagram (see Figure 8d). The onsets of both CL spectra at about 5.82 eV correspond to the band-edge emission of BNNTs, which are consistent with the result of ultraviolet–visible spectroscopy analysis. The broad luminescence peaks around 4.0 eV and 1.8 eV in both spectra could be attributed to the same intermediate band (designated as band *Ia*), while the peaks centered at 3.09 and 2.71 eV could be ascribed to another intermediate band (designated as band *Ib*). Luminescence peaks originating from these two intermediate bands are commonly observed in the luminescence spectra of multiwalled BN nanotubes and BN whiskers, but the origin of these two intermediate bands is still the subject of debates [47-49]. Since both types of BNNTs used in our study are grown under the same experimental conditions, we therefore expect that the impurities contained in both BNNTs are similar and the differences of the luminescence performance are primarily caused by structure differences. The emission peaks identified in the two CL spectra are basically the same except that the emission peak around 4.0 eV is dramatically broadened in the CL spectrum of bamboo-shaped BNNTs. This implies that band *Ia* in bamboo-shaped BNNTs is broader than in C-BNNTs. Therefore, band *Ia* most probably arises from structural defects since it is morphology dependent, while band *Ib* is safely attributed to chemical impurities since this band is almost the same for both BNNTs and is not affected by tube morphologies.

## Conclusions

In summary, we have developed a new approach to the fabrication of BNNTs with two morphologies using ammonia borane as a precursor. This method has the advantages of being simple and having a high yield. The structures and chemical compositions of the BNNTs are extensively characterized. The formation process of the BNNTs is interpreted by a two-stage growth mechanism. The luminescence performances of the BNNTs are investigated. It is found that the energy band gap of the BNNTs is independent of the tube morphologies. Luminescence peaks arising from the structural defects and chemical impurities have been distinguished. This method represents a facile path to large-scale synthesis of BNNTs with two morphologies, which can be used for future compact UV emitters.

## Supplementary Material

Additional file 1 Click here for file
