# Supplementary material for: Large-Scale Fabrication of Boron Nitride Nanotubes via a Facile Chemical Vapor Reaction Route and Their Cathodoluminescence Properties
Source: Nanoscale Res Lett. 2010 Sep 26;6(1):36. doi: 10.1007/s11671-010-9794-8 (PMC3248232; doi:10.1007/s11671-010-9794-8)
Supplement: Additional file 1 [file 1556-276X-6-36-S1.pdf]

Electronic Supplementary Information

Synthesis of boron nitride nanotubes via a facile chemical vapor reaction route and their cathodoluminescence properties

Bo Zhong[1], Xiaoxiao Huang[1], Guangwu Wen[1,2], Hongming Yu[1], Xiaodong Zhang[1], Tao Zhang[1] and Hongwei Bai[1]

[1] School of Materials Science and Engineering, Harbin Institute of Technology, Harbin 150001, People's Republic of China.

[2] School of Materials Science and Engineering, Harbin Institute of Technology (Weihai), Weihai 264209, People's Republic of China. Fax: +86-451-8641-3922; Tel: +86-451-8641-8860; E-mail: g.w.wen.hit@gmail.com

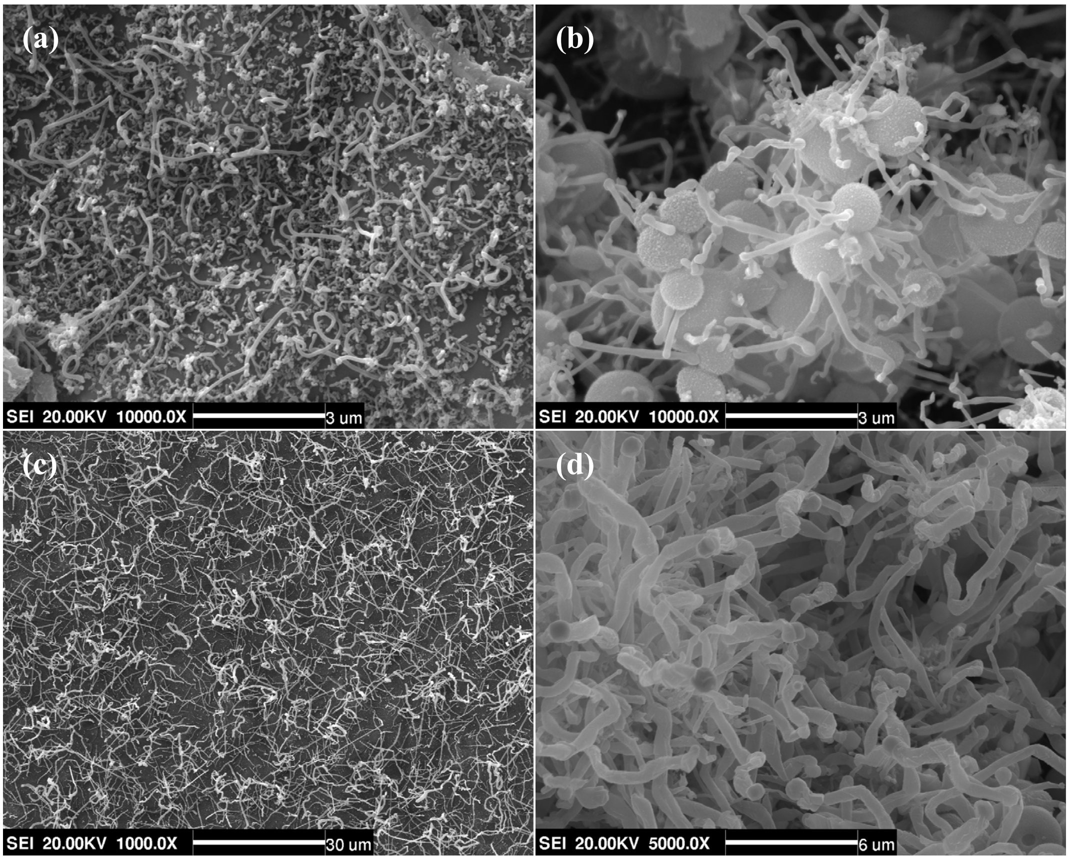

Figure S1. SEM images of BNNTs synthesized at different reaction temperatures: (a) 900 °C, (b) 1300 °C, (c) 1400 °C, (d) 1600 °C. Other experimental parameters are fixed and set as following: ammonia borane, 0.3g; ferrocene, 0.15 g; pressure, 0.8 MPa; gas, nitrogen; annealing time, 20 min.

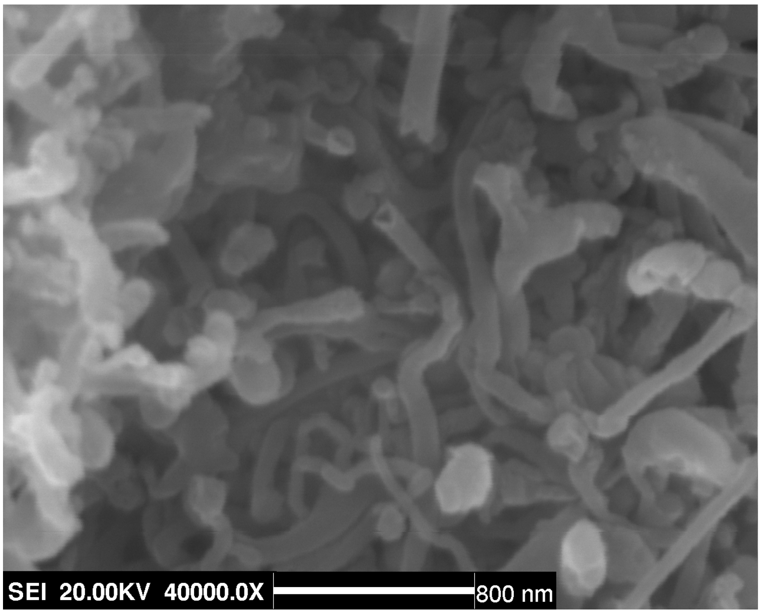

Figure S2. SEM image of BNNTs synthesized under argon atmosphere. Other experimental parameters are set as following: ammonia borane, 0.3g; ferrocene, 0.15 g; pressure, 0.8 MPa; temperature, 1600 °C; annealing time, 20 min.

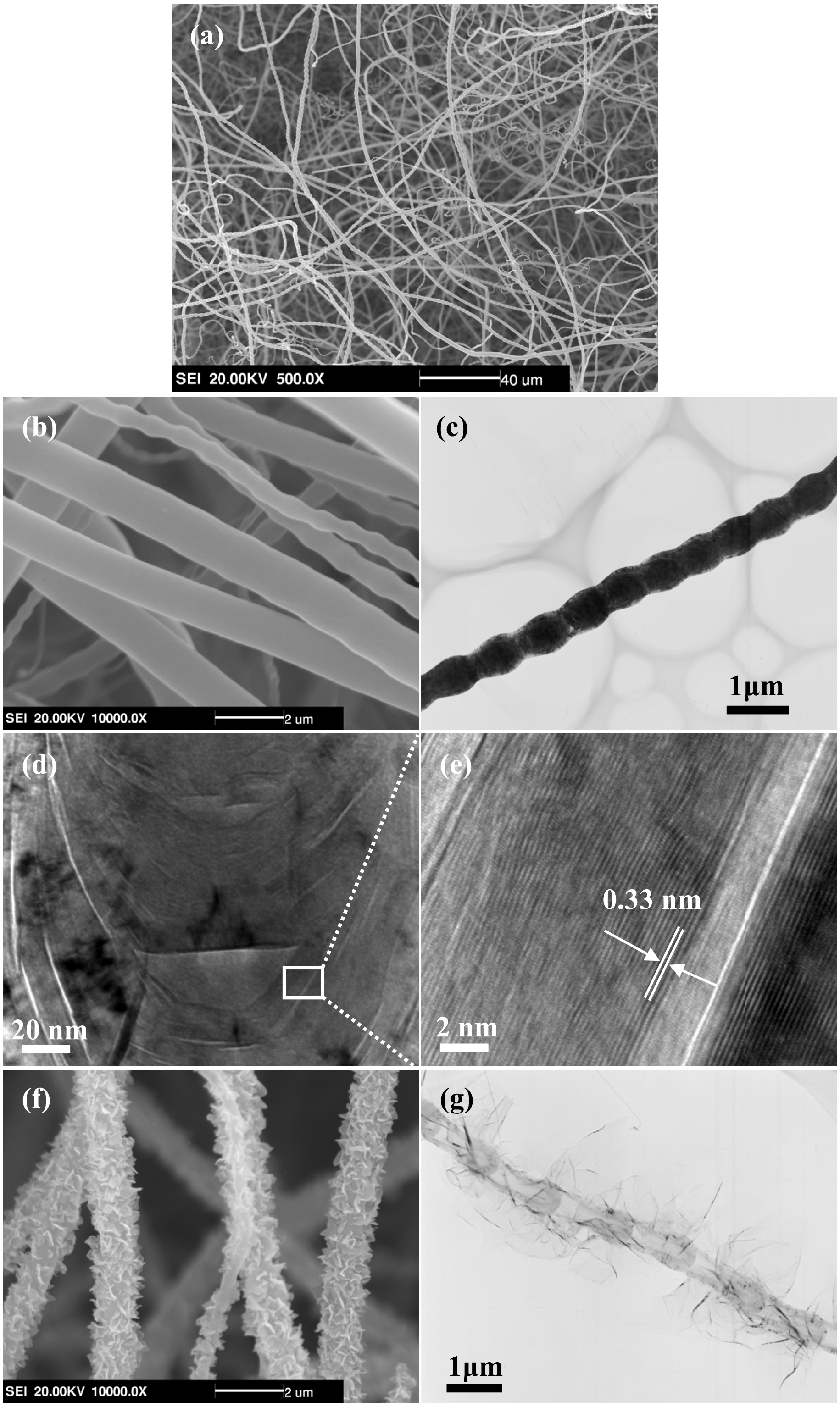

Figure S3. Typical SEM and TEM images of BNNTs synthesized using larger amount of ammonia borane (6.0 g) with other experimental parameters set as following: ferrocene, 1.5 g; gas, nitrogen; pressure, 0.8 MPa; temperature, 1400 °C; annealing time, 20 min. (a) Low magnification SEM image showing an overview of the BNNTs. High magnification SEM image (b), low magnification TEM image (c), High magnification TEM image (d) and HRTEM image of the

BNNTs with larger diameters, showing that when the amount of ammonia borane is increased, the diameters of the BNNTs are dramatically enlarged and the structures of the BNNTs are ultimately altered. SEM (f) and TEM (g) images of BNNTs with flakes extruding outwards.
